# Flucytosine and cryptococcosis: time to urgently address the worldwide accessibility of a 50-year-old antifungal

**DOI:** 10.1093/jac/dkt221

**Published:** 2013-06-20

**Authors:** Angela Loyse, Françoise Dromer, Jeremy Day, Olivier Lortholary, Thomas S. Harrison

**Affiliations:** 1 Cryptococcal Meningitis Group, Research Centre for Infection and Immunity, Division of Clinical Sciences, St. George's Hospital Medical School, London, UK; 2 Institut Pasteur, Unité de Mycologie Moléculaire, Centre National de Référence Mycoses Invasives et Antifongiques, Paris, France; 3 CNRS URA3012, Paris, France; 4 Oxford University Clinical Research Unit, Hospital for Tropical Diseases, Ho Chi Minh City, Vietnam; 5 Université Paris Descartes, Service des Maladies Infectieuses et Tropicales, Hôpital Necker-Enfants malades, Centre d'Infectiologie Necker Pasteur, IHU Imagine, Paris, France

**Keywords:** 5-FC, cryptococcal meningitis, cryptococcal meningitis treatment guidelines, access to essential antifungals for cryptococcal meningitis, 5-FC safety, combination antifungal therapy, opportunistic infection

## Abstract

Current, widely accepted guidelines for the management of HIV-associated cryptococcal meningoencephalitis (CM) recommend amphotericin B combined with flucytosine (5-FC) for ≥2 weeks as the initial induction treatment of choice. However, access to flucytosine in Africa and Asia, where disease burden is greatest, is inadequate at present. While research into identifying effective and well-tolerated antifungal combinations that do not contain flucytosine continues, an ever-increasing body of evidence from *in vitro*, *in vivo* and clinical studies points to the benefits of flucytosine in the treatment of CM in both intravenous combinations with amphotericin B and oral combinations with high-dose fluconazole. This article provides an up-to-date review of this evidence, and the current issues and challenges regarding increasing access to this key component of combination antifungal therapy for cryptococcosis.

## Introduction

### History

Flucytosine was first synthesized in 1957 as a potential anti-tumour agent.^1,2^ In 1963, murine studies demonstrated that flucytosine was effective against *Candida albicans* and *Cryptococcus neoformans*.^3^ Flucytosine was first used to treat human candidosis and cryptococcosis in 1968, and remains one of the oldest antifungal agents still in use.^4–6^ Flucytosine has some activity against dematiaceous fungi, including those causing chromomycosis,^7^ and against certain protozoa.^8^

### Structure and mechanism of action

**Figure 1.**
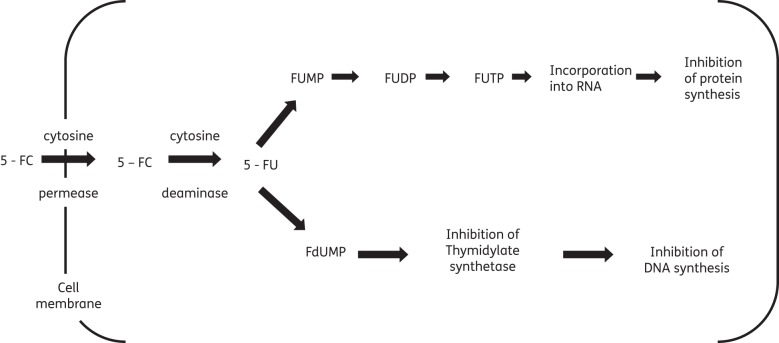
Intracellular pathway and mode of action of 5-fluorouracil (5-FU). Adapted with permission from Vermes *et al*.^6^ 5-FU is converted into 5-fluorouridine triphosphate (FUTP). FUTP alters the aminoacylation of tRNA through its incorporation into fungal RNA in place of uridylic acid, causing RNA miscoding and disturbed synthesis of proteins and carbohydrates. In addition, 5-FU is metabolized to 5-fluorodeoxyuridine monophosphate (FdUMP). FdUMP is a potent inhibitor of thymidylate synthetase, a key enzyme in the biosynthesis of DNA.

Flucytosine is a synthetic fluorinated analogue of cytosine.^1^ Flucytosine's antifungal activity derives from the rapid conversion of flucytosine into 5-fluorouracil (5-FU) within the cytosol of susceptible fungal cells.^9–11^ Flucytosine itself has no antifungal activity. The enzyme cytosine permease facilitates uptake of flucytosine into fungal cells. Cytosine deaminase then rapidly deaminates flucytosine to 5-FU.^12^ 5-FU is a potent antimetabolite that causes RNA miscoding and inhibits DNA synthesis through two separate mechanisms (Figure 1).^12,13^ Whether these two pathways are linked or independent, what their relative importance for the total antifungal effect of flucytosine is and what controls the intracellular fate of 5-FU remains unclear.^5^

Human cells do not possess the enzyme cytosine deaminase, unlike prokaryotic and fungal cells, and therefore cannot convert flucytosine into 5-FU.^14–17^ Flucytosine thus appeared to be an ideal drug, disturbing nucleic acid function exclusively in fungal cells. However, when flucytosine serum concentrations exceed 100 mg/L, patients experience symptoms of haematological and gastrointestinal toxicity characteristic of 5-FU chemotherapy.^14,15,18–20^ Furthermore, 5-FU catabolites have been detected in the urine and sera of patients at levels comparable to those found during 5-FU chemotherapy.^16,21^ Subsequently, *in vitro* studies have demonstrated that human intestinal microflora are capable of converting flucytosine into 5-FU, thus causing the bone marrow depression, hepatotoxicity, and gastrointestinal disturbance associated with flucytosine chemotherapy.^14,22,23^ In a recent Thai study comparing oral and intravenous formulations of flucytosine for the treatment of HIV-associated cryptococcosis, despite much lower concentrations of flucytosine detectable with oral flucytosine, 5-FU was detected in the serum of three patients on oral flucytosine versus one on intravenous flucytosine.^17^

### Pharmacokinetics and pharmacodynamics

The absorption of flucytosine is rapid in normal individuals, with bioavailability reported as 76%–89% after oral administration.^24^ However, the aforementioned Thai study indicated that the bioavailability of flucytosine, in common with a number of other drugs,^25^ may be reduced in late-stage HIV-infected patients.^17^ Indeed, the ratio of the AUC for oral and intravenous formulations suggested an oral bioavailability of only 45%.^17^

Flucytosine is a small, highly water soluble molecule that achieves good levels in tissue, cerebrospinal and vitreous fluids, and urine.^9,11,26^ Only 2.9%–4% of the drug is protein bound. Elimination of flucytosine is principally through the kidneys, flucytosine's plasma clearance being closely related to creatinine clearance.^24,26^ In patients with normal renal function, peak concentrations of flucytosine occur within 1–2 h of drug administration,^12,24^ and the half-life is 3–4 h.^24,26^ In patients with severe renal insufficiency, the half-life can increase up to 85 h,^24,26^ and the dosage of flucytosine must therefore be carefully adjusted in patients with renal impairment (Table 1).

**Table 1.** Flucytosine renal adjustment table

Creatinine clearance (mL/min)			
>40	20–40	10–20	<10
25 mg/kg po q6h	25 mg/kg po q12h	25 mg/kg po q24h	12.5 mg/kg po >q24h

Data from *in vitro*, animal model and some clinical studies suggests that flucytosine displays concentration-independent, time-dependent pharmacodynamics.^17,27–29^

### In vitro studies

Standardized methods for testing flucytosine susceptibility include modifications of the CLSI M27-A protocol for susceptibility testing of yeasts^30^ and broth microdilution techniques.^31^ However, data regarding appropriate breakpoints for defining flucytosine susceptibility in *C. neoformans* are limited.^31,32^ Rex and Pfaller^31^ have suggested that breakpoints similar to those used for *Candida* species may be considered for *C. neoformans*: *C. neoformans* isolates with MICs ≤4 mg/L are fully susceptible, isolates with MICs between 4 and 16 mg/L have intermediate susceptibility, whereas isolates with MICs >16 mg/L are presumed to be flucytosine resistant.^33^

In a multicentre study, the *in vitro* antifungal susceptibility of *C. neoformans* to fluconazole, amphotericin B (AmB) and flucytosine failed to predict early clinical outcome in cryptococcosis patients.^34^ Different techniques used to determine antifungal susceptibility include the CLSI method, Etest and broth microdilution in yeast nitrogen base (YNB) medium.^34^ A lack of correlation between *in vitro* antifungal susceptibility tests and early patient clinical outcome may be explained by the small number of high MIC isolates.^31,34^

### Resistance

The estimated prevalence of primary flucytosine-resistant *C. neoformans* is 1%–2%, although incidences of up to 7% have been reported.^35–42^ *In vitro* resistance to commonly used antifungal agents, including flucytosine, has not increased in the UK or the USA.^39,40,43^ In the USA, the rate of *C. neoformans* resistance to flucytosine ranged from 1.6% in 1992–1994 isolates to 2.2% in 1996–1998 isolates.^39^ Published data regarding antifungal resistance are limited in geographical scope and only cover short time spans.

Pfaller *et al*.^40^ addressed this limitation in the data by conducting a multicentre antifungal susceptibility study of 1811 *C. neoformans* isolates obtained from 100 medical centres in five geographic regions (Africa, Europe, Latin America, North America and the Pacific region) over 15 years. Isolates were submitted to a central reference laboratory and tested using the CLSI (formerly NCCLS) broth dilution method.^40^ High-level resistance to flucytosine (MIC ≥32 mg/L) remained essentially unchanged at 1%–2% over the entire study period,^40^ consistent with results from smaller studies from Spain, Egypt and South America.^41,42,44^ However, interestingly, full susceptibility to flucytosine (MIC ≤4 mg/L) ranged from 35% in North America to 68% in Latin America.^40^ North American isolates were considerably less susceptible to both flucytosine and fluconazole compared with other geographical regions,^40^ although susceptibilities in North America isolates increased over time (for flucytosine, from 34% in 1990–1994 to 66% in 2000–2004).^40^ The reason for this geographical and temporal difference in full flucytosine and fluconazole susceptibility remains unclear. It is possible that some isolates were included from relapse cases and that the improvement in susceptibility may have reflected a decrease in such cases and overall drug pressure of flucytosine and fluconazole with introduction of antiretroviral therapy.^40^

Two potential mechanisms can cause flucytosine resistance: (i) mutations leading to deficiencies in the enzymes required for cellular uptake or metabolism of flucytosine (i.e. cytosine permease and deaminase)^6,20,45^ and (ii) increased synthesis of pyrimidines that compete with the fluorinated antimetabolites of flucytosine.^6,20^

Secondary resistance precludes the use of flucytosine as a single agent.^45,46^ *In vitro* studies of flucytosine and other antifungals including AmB suggest the ability to overcome flucytosine resistance may depend on the mechanism of resistance.^35,45^ If resistance is due to a defective cytosine permease, it may be overcome by a drug such as AmB that facilitates flucytosine cellular uptake.^35^

Indeed, it has been shown that synergy *in vitro* between AmB and flucytosine can occur even where there is evidence of resistance to flucytosine.^35,47^

## Experimental cryptococcosis

Flucytosine, alone and in combination, has been studied in murine and corticosteroid-treated rabbit models of cryptococcosis. In murine models, AmB plus flucytosine demonstrates additive or synergistic interactions.^48–51^ The combination of AmB (0.5 mg/kg/day) and flucytosine (250 mg/kg/day) was significantly more effective than either AmB or flucytosine monotherapy for reducing fungal burden in brain and spleen for both flucytosine-susceptible and flucytosine-resistant isolates in a murine model of disseminated cryptococcosis.^48^ Combination therapy with a reduced dosage of flucytosine at 100 mg/kg/day and AmB at 0.5 mg/kg/day was still superior to monotherapy for reducing the fungal burden in the brain, but not the lungs or spleen, for the flucytosine-resistant isolate.^48^

In contrast to AmB/flucytosine, a beneficial interaction between azoles and flucytosine in animal models is less evident. While the combination of fluconazole and flucytosine appears beneficial overall in murine models of cryptococcosis,^52–55^ one study of a rabbit model of meningeal cryptococcosis showed evidence of a dose–response with fluconazole, but no *in vivo* benefit, in terms of reduced cryptococcal cfu counts in the CSF with the addition of flucytosine to low-dose fluconazole.^56^ It is worth noting however, as discussed below, that this flucytosine–fluconazole interaction, and the nature of drug interactions in general, may depend critically on the concentration of the component drugs used. Earlier studies of combinations of flucytosine with itraconazole and ketoconazole have also demonstrated indifferent interactions.^49,50^

Finally, combinations of all three available agents have been studied: a study in mice evaluated the antifungal efficacy of AmB colloidal dispersion (ABCD) combined with flucytosine with or without fluconazole,^52^ using regression methods for estimating and visualizing the dose–response surfaces for survival, weight loss and brain cfu counts. The combination of ABCD and flucytosine achieved a 100% survival rate, however, the addition of fluconazole was required to prevent weight loss (*P *< 0.001) and to achieve maximal antifungal effect (*P* < 0.001).^52^ There was a strong association between the numbers of cfu recovered per gram of brain tissue and the dosages of both ABCD and fluconazole (*P* < 0.001). A moderate association (*P* < 0.01) was seen with the dosage of flucytosine. Consistent with concentration-independent activity for flucytosine, maximal fungicidal effect was seen with high ABCD (5.0–7.5 mg/kg) and fluconazole (≥30 mg/kg/day) doses but a moderate flucytosine dose (20–60 mg/kg/day).^52^

## Cryptococcosis in clinical studies

### Flucytosine monotherapy

Rapid onset of resistance precludes the use of flucytosine monotherapy. Hospenthal and Bennet^57^ described the treatment of 27 HIV-negative patients with disseminated cryptococcal disease given flucytosine monotherapy when the drug first became available. Twenty-three patients received either 4 g or 6 g flucytosine daily, with four patients receiving 7–10 g flucytosine daily. The initial dosing selection (4 g/day) was based on the historically approved dose. One-third of patients achieved long-term cure with flucytosine monotherapy. However, secondary flucytosine resistance occurred in isolates from ∼50% patients who did not respond to therapy or relapsed. Flucytosine was nonetheless well tolerated, even at high doses and for prolonged treatment courses, with only infrequent and mild toxicity reported.^57^

### Flucytosine and amphotericin B therapy (Table 2)

Clinical trials prior to the HIV epidemic support the use of flucytosine in combination with AmB. In a randomized trial published in 1979, 66 HIV-negative patients were randomized to either low-dose AmB (0.4 mg/kg/day) for 10 weeks or AmB (0.3 mg/kg/day) plus flucytosine (150 mg/kg/day) for 6 weeks. In the subset of 50 patients considered to be adherent, combination therapy cured or improved 67% of patients compared with 41% of AmB monotherapy patients, although this result was not statistically significant.^58^ Fewer treatment failures or relapses (3 versus 11), more rapid CSF sterilization (*P* < 0.001) (*P* = 0.05 if non-adherent patients were included in this analysis) and less nephrotoxicity (*P* < 0.05) were noted in the combination arm.^58^ However, unsurprisingly, given the small patient numbers, no mortality difference was detected.^58^ Adverse reactions to flucytosine occurred in 11 of 34 patients, but none was considered life threatening.^58^

**Table 2.** Table of key AmB + flucytosine-containing antifungal therapy randomized controlled trials

Study	Year	No. of patients	Treatment arms	Treatment duration	Median time to CSF sterilization (days)	Deaths at 10 weeks (%)	Role of 5FC/AmB combination therapy
Bennett^58^ (HIV negative patients)	1979	51	AmB 0.4 mg/kg	10/52		no difference in treatment arms	less nephrotoxicity (*P* < 0.05) and fewer treatment failures/relapses (3 versus 11)
			AmB 0.3 mg/kg + 5-FC 150 mg/kg	6/52	more rapid CSF sterilization (*P* < 0.001)		
Larsen^89^	1990	20	AmB 0.7 mg/kg + 5-FC 150 mg/kg	10/52	16	0 (0/6)	superior mycologic and clinical efficacy
			FLU 400 mg	10/52	41 (*P* = 0.02)	28.5 (4/14)	
van der Horst^60^	1997	306	Step 1^a^: AmB 0.7 mg/kg	2/52	≤14, 51% versus 60%; *P* = 0.06	9.4	not receiving 5-FC during initial 2/52 = factor associated with CM relapse (RR 5.88, *P* = 0.004) (Reich *et al*.^78^)
Saag^61^			Step 2^a^: AMB 0.7 mg/kg + 5-FC 100 mg/kg	2/52			addition of 5-FC during initial 2/52 of treatment independently associated with CSF sterilization
Brouwer^62^	2004	64	AmB 0.7 mg/kg	2/52	< 14	22	AmB + 5-FC most rapidly fungicidal regimen
			AmB 0.7 mg/kg + 5-FC				
			AmB 0.7 mg/kg + FLU 400 mg				
			AmB 0.7 mg/kg + 5-FC + FLU 400 mg				
Bicanic^63^	2008	64	AmB 0.7 mg/kg + 5-FC	2/52	not reported	24	superior mycologic efficacy of higher dose AmB
			AmB 1 mg/kg + 5-FC				
Loyse^67^	2011	80	AmB 0.7–1 mg/kg+ 5-FC	2/52	not reported	29	no significant difference in EFA between four treatment arms
			AmB 0.7–1 mg/kg+ FLU 600 mg twice daily				
			AmB 0.7–1 mg/kg+ FLU 800 mg/day				
			AmB 0.7–1 mg/kg+ VCZ 300 mg twice daily				
Day^66^	2011	298	AmB 1 mg/kg	4/52	not reported		mortality benefit with 2/52 AmB + 5-FC compared with AmB monotherapy at 2/52. Benefit at 6 months of AmB + 5-FC versus AmB + FLU
			AmB 1 mg/kg + 5-FC 100 mg/kg	2/52			
			AmB 1 mg/kg + FLU 400 mg twice daily				

5-FC, 5-fluorocytosine; FLU, fluconazole; AmB, amphotericin B; VCZ, voriconazole.

^a^These references represent two steps of the same trial.

A follow-up randomized trial of 91 HIV-negative patients compared 4 versus 6 weeks of AmB (0.3 mg/kg/day) and flucytosine (150 mg/kg/day). Six weeks was associated with an 85% cure or improvement rate (16% relapse rate) versus a 75% cure or improvement rate (27% relapse) after 4 weeks of treatment.^59^ Toxicity was reported as significant in both groups (44% versus 43%).^59^ When comparing early flucytosine serum levels of 38 patients who developed flucytosine toxicity with the levels of 47 patients without toxicity, the development of toxic effects correlated significantly with the presence of serum flucytosine concentrations ≥100 mg/L for 2 or more weeks (*P* = 0.005).^59^ It is important to emphasize that low-dose AmB in combination with high-dose flucytosine was used in both these early studies, and fluconazole was unavailable.

In the era of the HIV epidemic, more recent trials support AmB-flucytosine combination therapy. In a landmark trial of higher dose AmB (0.7 mg/kg) and lower dose flucytosine (100 mg/kg), 2 weeks of AmB monotherapy was compared with 2 weeks of AmB-flucytosine combination therapy.^60^ A trend towards increased CSF sterilization at 2 weeks was noted with combination therapy (60% versus 51% for AmB alone, *P* = 0.06),^60^ and, in a multivariate model, the addition of flucytosine was independently associated with 2 week CSF sterilization (OR 1.92, 95% CI 1.15, 3.22, *P* = 0.01).^60^ Not receiving flucytosine during the initial 2 weeks of induction treatment was the factor most strongly associated with cryptococcal meningitis (CM) relapse at a time when no highly active antiretrovirals were available [relative risk (RR) 5.9, *P* = 0.004].^61^

A randomized trial of 64 HIV-infected CM patients confirmed that AmB (0.7 mg/kg) plus flucytosine (100 mg/kg/day) was the most rapidly fungicidal regimen when compared with AmB alone, AmB plus fluconazole (400 mg/day) or even triple therapy (AmB plus flucytosine plus fluconazole 400 mg/day).^62^ The trial used a new endpoint, rate of clearance of infection, or early fungicidal activity (EFA), based on quantitative CSF cultures. EFA has subsequently proved to be a powerful tool to determine the relative fungicidal activity of novel antifungal regimens, and in a combined cohort of >500 patients, was independently associated with mortality at 2 and 10 weeks, alongside altered mental status at presentation and high baseline fungal burden.^63–65^

**Figure 2.**
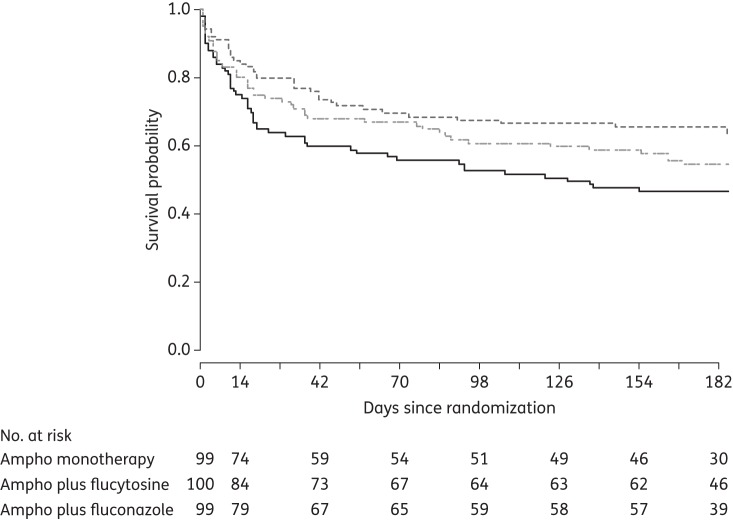
Survival curves, by treatment group, for patients with HIV-associated cryptococcal meningitis in Vietnam treated with AmB (1 mg/kg/day) (Arm I) alone for 4 weeks (continuous line), with 5-FC (100 mg/kg/day) (Arm II) for 2 weeks (black dashed line) or with fluconazole (800 mg/day for 2 weeks) (grey dotted/dashed line), followed by fluconazole in all three arms. HR (95% CI) at 14/7, II versus I: 0.57 (0.30, 1.08), *P* = 0.08; III versus I: 0.71 (0.45, 1.11), *P* = 0.13; at 70/7, II versus I: 0.61 (0.39, 0.97), *P* = 0.04; III versus I: −0.78 (0.44, 1.41), *P* = 0.42. From Day JN, Tran TTH, Wolbers M *et al.* *N Engl J Med* 2013;**368**: 1291–302. Copyright Massachusetts Medical Society 2013. Reprinted with permission.^66^

In an important Phase III study, 298 patients with a first episode of CM were randomized to three induction treatment arms: 4 weeks of AmB (1 mg/kg/day) alone, AmB + flucytosine (100 mg/kg/day) for 2 weeks or AmB + fluconazole (400 mg twice daily) for 2 weeks. For the first time, a mortality benefit was seen with the addition of flucytosine compared with AmB monotherapy [hazard ratio (HR) 0.57, 95% CI 0.30, 1.08, *P* = 0.08 at 2 weeks; HR 0.61, 95% CI 0.39, 0.97, *P* = 0.04 at 10 weeks] (Figure 2).^66^ There was no statistical difference in terms of mortality between AmB plus flucytosine and AmB plus fluconazole at the 2 and 10 week primary endpoints,^66^ although an adjusted analysis at 6 months found higher mortality with fluconazole (HR 1.81, 95% CI 1.14, 2.88, *P* = 0.01). The rate of clearance of infection (log_10_ cfu/mL/day) was also greater for AmB plus flucytosine (HR −0.42, 95% CI −0.44, −0.40) compared with both AmB monotherapy (HR −0.31, 95% CI −0.34, −0.29) and AmB plus fluconazole (HR −0.32, 95% CI −0.34, −0.29).^66^ The study confirms the clinical as well as mycological superiority of AmB plus flucytosine over AmB alone. Whether AmB plus high-dose fluconazole is a reasonable alternative to AmB plus flucytosine remains an open question, and an important one, given the current free availability of fluconazole and the difficulties of obtaining flucytosine in Africa. In terms of clearance of infection, a recent Phase II study found no significant difference in EFA between AmB plus flucytosine and AmB plus fluconazole at either 800 or 1200 mg/day.^67^

In a prospective cohort study of 230 patients (77% HIV positive), the Crypto A/D study, 106 (46%) patients received flucytosine as part of induction therapy for a median of 12.3 ± 4.3 days (for HIV-positive patients).^68,69^ The lack of flucytosine therapy during the induction treatment was independently associated with mycological failure at 2 weeks (OR 3.8, 95% CI 1.9, 7.8]).^68^ Despite more severe infections in this group, mycological failure at 2 weeks was significantly less frequent among patients treated with AmB plus flucytosine than any other regimen (mainly AmB or fluconazole monotherapy) [20/86 (23%) versus 47/100 (47%), *P* < 0.001].^69^ Treatment failure (death or mycological failure) in patients with meningoencephalitis and abnormal neurology was also less frequent with AmB plus flucytosine than any other regimen [10/40 (25%) versus 26/36 (72%), *P* < 0.001], and prescription of flucytosine for <14 days was independently associated with treatment failure at 3 months (OR 3.30, 95% CI 1.12, 9.70, *P* = 0.03).^69^

### Flucytosine and fluconazole combination therapy (Table 3)

Clinical studies consistently support the addition of flucytosine to high-dose (800–1200 mg/day) fluconazole for the treatment of CM. In a prospective, open-label cohort study, 32 patients with HIV-associated CM were treated with oral fluconazole (400 mg/day) and flucytosine (150 mg/kg/day) for a total of 10 weeks.^70^ Sixty-three per cent of the patients completed 10 weeks of oral treatment with a negative CSF culture, and the median time to CSF sterilization was 23 days. However, side effects were significant enough to lead to flucytosine withdrawal in nine patients (28%).^70^ This relatively high rate of flucytosine discontinuation is most likely related to both the high dose and long course of flucytosine used. Indeed, 95% of patients tolerated flucytosine for at least 2 weeks.^70^

**Table 3.** Table of key flucytosine and fluconazole combination therapy randomized controlled trials

Study	Year	No. of patients	Treatment	Treatment duration	Median time to CSF sterilization (days)	Deaths at 10 weeks (%)	Role of 5-FC/combination AmB + 5-FC therapy elucidated
Larsen^70^	1994	32	FLU 400 mg + 5-FC 150 mg/kg	10/52	23	13	Rate of clinical success at 10/52 greater than reported with either FLU or AmB monotherapy
Mayanja-Kizza^71^	1998	58	FLU 200 mg + 5-FC 150 mg/kg (2/52)	total 10/52	>60	50	6/12 survival rate in combination therapy arm significantly higher comparing with monotherapy (32% vs 12%, *P* = 0.022)
			FLU 200 mg			65	
Milfechik^72^	2008	34	FLU 800–2000 mg (dose escalation)	Total 10/52 FLU/5-FC for initial 4 weeks	Increasing FLU dosages increased survival and reduced time to CSF sterility	75% ‘success rate’	Addition of 5-FC to FLU improved overall response rates (*P* < 0.02, log rank test) at each dose level of FLU except at 1600 mg dosing
			FLU 800–1200 mg + 5-FC (100 mg/kg)				
Nussbaum^64^	2010	41	FLU 1200 mg	2/52		37	EFA significantly higher and mortality lower than for FLU alone
			FLU 1200 mg + 5-FC (100 mg/kg)	2/52		10	

5-FC, 5-fluorocytosine; FLU, fluconazole; AmB, amphotericin B.

In a randomized trial in Uganda, a combination of very-low-dose fluconazole (200 mg/day) and flucytosine (150 mg/kg/day for the first 2 weeks) was compared with low-dose fluconazole monotherapy (fluconazole 200 mg/day for 2 months) in 58 patients. The survival rate with combination treatment, although disappointing, was significantly higher than with fluconazole monotherapy (32% versus 12%, *P* = 0.02, at 6 months).^71^

In a Phase II dose escalation study by Larsen *et al.*,^72^ 89 patients with a first episode of HIV-associated CM were treated with 800–2000 mg/day fluconazole administered alone for 10 weeks or in combination with flucytosine (100 mg/kg/day) for the first 4 weeks. A dose–response effect was seen with increasing fluconazole dose, at least up to 1600 mg/day. The addition of flucytosine to fluconazole improved the overall response rates (*P* < 0.02, log rank test), especially at the 800 and 1200 mg/day fluconazole dosages.^72^ Overall success, defined as being alive with a negative CSF culture on or before 10 weeks, was 75% for subjects who received fluconazole and flucytosine in combination.^72^

**Figure 3.**
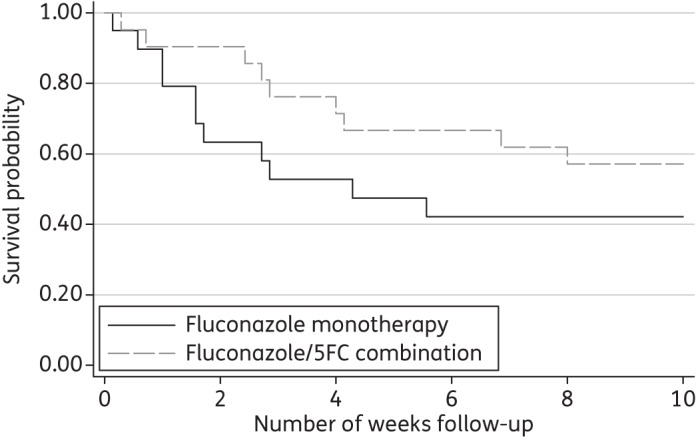
Survival curves, by treatment group for patients with HIV-associated cryptococcal meningitis in Malawi treated with fluconazole 1200 mg/day or fluconazole 1200 mg/day plus flucytosine 100 mg/kg/day for the initial 2 weeks. One patient lost to follow up was censored. *P* = 0.05 at 2 weeks and *P* = 0.25 at 10 weeks by Cox regression. Reproduced with permission from Day *et al*.^6,4^

Given evidence for the safety and efficacy of higher dose fluconazole,^72,73^ a more recent randomized trial in Malawi of 44 HIV-seropositive patients compared the EFA of fluconazole 1200 mg/day versus fluconazole 1200 mg/day plus flucytosine 100 mg/kg/day.^64^ The EFA for the combination arm was significantly higher than for fluconazole alone (−0.28 ± 0.17 log cfu/mL/day versus 0.11 ± 0.09 log cfu/mL/day, *P* = 0·001). In addition, there were fewer deaths in the combination arm that almost reached statistical significance at 2 weeks, despite the small size of the study (Figure 3).^64^ Combination therapy was well tolerated despite more episodes of neutropenia (five versus one, grade III and IV within the first 2 weeks of antifungal therapy), which were rarely treatment limiting and not associated with increased evidence of infection.^64^ While trials can only be compared with caution, the EFA of this combination of fluconazole with flucytosine (−0.28 log cfu/mL/day) is the closest an oral antifungal regimen has come to the fungicidal activity of an AmB monotherapy regimen (−0.31 log cfu/mL/day for AmB 0.7 mg/kg alone in Thailand).^62,64^ The large randomized comparative Phase III ACTA study will assess whether the oral fluconazole + flucytosine combination is as effective as the recommended AmB + flucytosine or AmB + fluconazole strategy for induction treatment of HIV-associated CM [ISRCTN: 45035509].

### Safety of flucytosine

The most important side effect of flucytosine is bone marrow depression, particularly neutropenia; other side effects include hepatotoxicity, diarrhoea and vomiting. Bone marrow depression and hepatotoxicity are associated with prolonged high serum flucytosine concentrations, generally >100 mg/L, and are thought to be mediated by 5-FU.^6,58^ Flucytosine is a category C drug in pregnancy and is teratogenic in rat animal models.

Earlier clinical trials, in which flucytosine toxicity was significant, used high-dose flucytosine (150 mg/kg/day) for prolonged durations.^58,59,70^ Reported toxicity has been significantly less in recent trials using shorter courses of lower dose flucytosine (100 mg/kg/day).^17,60,62,67,74^ Milefchik *et al.*^72^ reported grade 4 neutropenia in 18% of patients given flucytosine (100 mg/kg/day) for 4 weeks, without evidence of increased infections. In the van der Horst MSG/ACTG study there was a 3% rate of drug discontinuation in the first 2 weeks with 2 weeks flucytosine, equally split between 202 patients receiving and 179 not receiving flucytosine, and almost all clearly AmB-related, with no discontinuations due to neutropenia.^60^ Similarly in Thailand, 2 weeks AmB plus flucytosine (100 mg/kg/day) was well tolerated: there were no incidences of grade 4 neutropenia and no drug discontinuations in the 2 weeks of combination therapy.^17,62^ Overall, in EFA studies in Africa and Asia, flucytosine at 100 mg/kg/day for 2 weeks with either AmB or fluconazole has been associated with grade 4 neutropenia in 8/183 (4.4%) patients.^17,62,64,67,74–76^ It is important to note that in all these studies with 2 week flucytosine courses, including that of the MSG/ACTG, full blood counts and renal function tests, not flucytosine drug levels, were used to monitor therapy. Nevertheless, current US guidelines recommend monitoring serum flucytosine levels in patients receiving flucytosine after 3–5 days of therapy, aiming for 2 h post-dose serum flucytosine levels of 30–80 mg/L,^77^ to avoid toxicity and prevent emergence of resistance. Where serum flucytosine level monitoring is not available, bone marrow and renal function should be monitored frequently and flucytosine dose adjustment made with the aid of a nomogram (Table 1).^77^ Studies in resource-poor settings have demonstrated that flucytosine may be used safely and effectively without flucytosine serum level monitoring for up to 2 weeks, as long as haematological and renal function are monitored closely, flucytosine dosage adjustments are made as required and AmB nephrotoxicity is minimized through saline and fluid loading.^60,62,64,67,74–76^ If grade 4 cytopenias occur, flucytosine should be withheld. Of note, in Africa many patients have relatively low neutrophil counts^78^ and not infrequently in the course of monitoring, counts, being variable, may cross the grade 3 threshold but recover spontaneously with repeat testing.

The reason for the reduced side effects seen with flucytosine at 100 mg/kg/day for 2 weeks in studies of HIV-associated cryptococcal infection, and why flucytosine drug level monitoring may not be essential in this particular setting, may be found in a study of the pharmacokinetics and dynamics of oral versus intravenous flucytosine.^17^ The bioavailability of oral flucytosine in HIV-infected patients with CM was only ∼50%, and flucytosine levels, although sufficient to remain above the MIC and therefore maintain efficacy, were well below those usually associated with toxicity.^17^ Indeed, 5-FU was infrequently detected in serum.

### Guidelines

Based on the evidence outlined above, current treatment guidelines are consistent in recommending inclusion of flucytosine in initial combination therapy for CNS and severe non-meningeal cryptococcal infection. Current Infectious Diseases Society of America (IDSA) and WHO guidelines for the management of HIV-associated CM recommend AmB (0.7–1 mg/kg/day) plus flucytosine (100 mg/kg/day) orally in four divided doses for at least the first 2 weeks as the induction treatment of choice;^77,79^ and, while the recommended AmB formulation and duration of induction varies, inclusion of flucytosine applies across all host groups: HIV-infected, transplant (lipid formulations of AmB plus flucytosine), non-HIV non-transplant (induction for 4–6 weeks) and children. Only in pregnancy should flucytosine use be carefully weighed as part of a risk–benefit analysis.

In resource-limited settings, without access to or facilities to safely give AmB, IDSA guidelines suggest fluconazole at at least 1200 mg/day plus flucytosine, if it is available, as one option for the initial 2weeks.^77^ Advice from the WHO also advocates inclusion of flucytosine in both AmB and high-dose fluconazole-based combinations, with the proviso that fluconazole should be used as an alternative second drug with AmB in the many settings where flucytosine is currently not available.^79^

Implementation of these recommendations for optimal treatment in resource-limited areas in Africa and Asia with the highest burden of cryptococcal disease is infrequent, however, and will remain so until and unless access to flucytosine is widened.

## Access to flucytosine

In the context of the HIV epidemic, CM is the leading cause of community-acquired meningitis in sub-Saharan Africa, causing an estimated 500 000 deaths annually in this region.^80^ CM mortality rates remain unacceptably high. In Africa, 10 week mortality ranges from 24% to >60%.^73,76,81–84^ Widening access to optimal antifungal therapy requires urgent action if this high associated mortality is to be reduced to the lower levels reported from developed country settings (10 week mortality 10%–26%).^60,68^ Improving access to essential antifungals must be achieved alongside efforts to treat patients earlier through improved diagnostics^85^ and effective management of CM complications such as raised intracranial pressure.^86^

Meda Pharma Pharmaceuticals (France) is the manufacturer of flucytosine. Recently a new equivalent formulation of oral flucytosine produced by Sigmapharm Labs LLC (USA) has been approved by the FDA. Flucytosine is manufactured by a number of companies in China, Taiwan and India, although the bioequivalence of these alternate sources of flucytosine is currently unknown. However, flucytosine is currently unavailable and unregistered in most of Africa and Asia, where the disease burden is greatest, despite flucytosine being a simple nucleotide analogue that has been off patent for many years.^87,88^ The registration of flucytosine in South Africa, where flucytosine was previously marketed by Roche, lapsed in 1996.^87,88^ Even where flucytosine is available, it is often not administered because of overstated fears regarding toxicity, or because the severity of cryptococcal disease is underestimated. Poor demand coupled with a lack of precise disease burden data in Africa and Asia have contributed to market failure for flucytosine. Given the current high cost of flucytosine due to a lack of competition, generic manufacturers targeting low- and middle-income countries are urgently needed. In addition, given the current four times daily dosing, a slow release formulation would be of great benefit, and should remain equally effective given the concentration-independent pharmacodynamics of flucytosine.

As the evidence for the benefit of flucytosine-containing combination therapy for cryptococcosis continues to mount, improving access to flucytosine in sub-Saharan Africa and Asia should become a key achievable component of efforts to reduce the global mortality burden from HIV-associated cryptococcal infection. Coordinated efforts from governmental and international stakeholders are required to disseminate and implement current treatment guidelines, encourage generic flucytosine production, facilitate flucytosine registration, and widen access to this key component of combination antifungal therapy for cryptococcal meningitis.

## Transparency declarations

O. L. has received grants or speaker's fees from Gilead Sciences, Merck, Astellas and Pfizer. All other authors: none to declare.

### Contributions

A. L. wrote the manuscript. F. D., O. L., J. D. and T. S. H. contributed equally to editing the manuscript.
